# COVID-19 and visitation to Central Park, New York City

**DOI:** 10.1371/journal.pone.0290713

**Published:** 2023-09-13

**Authors:** Weizhe Weng, Lingxiao Yan, Kevin J. Boyle, George Parsons

**Affiliations:** 1 Food and Resource Economics Department, University of Florida, Gainesville, Florida, United States of America; 2 National School of Agricultural Institution and Development, South China Agricultural University, Guangzhou, Guangdong, China; 3 Pamplin College of Business, Virginia Tech, Blacksburg, Virginia, United States of America; 4 School of Marine Science and Policy, University of Delaware, Newark, Delaware, United States of America; US Government Accountability Office, UNITED STATES

## Abstract

Central Park is an iconic feature of New York City, which was the first and one of the hardest hit cities in the United States by the Coronavirus. State-level stay-at-home order, raising COVID-19 cases, as well as the public’s personal concerns regarding exposure to the virus, led to a significant reduction of Central Park visitation. We utilized extensive cellphone tracking data to conduct one of the pioneering empirical studies assessing the economic impact of COVID-19 on urban parks. We integrated the difference-in-difference (DID) design with the recreation-demand model. The DID design aids in identifying the causal impacts, controlling for unobservable factors that might confound the treatment effects of interest. Concurrently, the recreational demand model examines the driving factors of visitation changes and enables us to estimate the welfare changes experienced by New York City’s residents. Our findings shine a light on the substantial, yet often overlooked, welfare loss triggered by the pandemic. The analysis indicates that the pandemic resulted in a 94% reduction in visitation, corresponding to an annual consumer surplus loss of $450 million. We noted a rebound in visitation following the initial outbreak, influenced by shifts in government policy, weather conditions, holiday periods, and personal characteristics.

## Introduction

The Coronavirus (COVID-19) pandemic has posed unprecedented challenges for societies worldwide. In response to the public health threat, governments at different levels around the world declared various lockdown and social-distancing policies to slow the spread of the disease. These measures have inevitably impacted the use of public spaces, including urban parks, by restricting travel or limiting visitation. However, given that viruses are less transmissible outside, outdoor recreational activities, such as visits to urban parks, may have served as crucial outlets for people to alleviate the stress induced by the pandemic.

The question of how the pandemic might have affected residents’ visitation to urban parks remains open. On one hand, the pandemic, coupled with government restrictions, may have influenced people’s personal decisions about traveling and spending time in public areas. This could have led to voluntary avoidance behavior, consequently decreasing park visitation [1]. Conversely, the outdoors is considered a much lower risk setting for COVID-19 transmission than indoors. Outdoor recreation activities, therefore, could offer a safe alternative to navigate the risks and stresses associated with the pandemic. Given their proximity to residential and work areas, urban parks may have provided a welcome break from the monotony of home-based living and working routines, potentially leading to increased park visitations.

Several studies have conducted large spatial-scale investigations of park visitation during the COVID-19, while having mixed findings. A national study in the U.S., examining the impact of COVID-19 on recreational trips and found decreased participation and economic value losses [2]. Another study explored visitation trends to green spaces in England, noting a shift in visitation to accessible greenspaces during the lockdown [3]. Additionally, an analysis of the effects of COVID-19 and governmental responses to the pandemic on park visitation, discovering that lockdown and social-distancing policies correlated with increased park visits [4]. A further examination of visitation patterns at six major U.S. national parks, revealing a surge in visitation following the easing of restrictions [5].

In this study, we investigate the impacts of COVID-19 on visitation to a specific urban park, Central Park in New York City (NYC), U.S. We provide empirical estimates of consumer surplus changes using the travel cost method. Estimating the economic values of urban parks is essential for park management and environmental planning [6]. In the event of a pandemic, understanding the scale of economic impacts can help policymakers identify associated losses and propose potential remedial solutions. However, due to the public goods feature of urban parks, there is no market price for them. Instead, we should rely on non-market valuation tools, such as the travel cost model, to estimate the associated values and the welfare changes [7].

Located in one of the world’s most densely populated and affluent cities, Central Park holds substantial aggregate recreational use value. The impact of COVID-19 on visitation, however, is multifaceted. Central Park may have been appealing for visitation and social distancing due to its size and the variety of facilities it offers. Meanwhile, NYC suffered the brunt of the initial onslaught of the COVID-19 pandemic in the U.S., which could have dampened visitation. The governor of New York declared a state of emergency, one of the actions of which was to limit park access to ensure social distancing [8].

By leveraging big cellphone data, we gained access to the weekly visitation patterns of NYC residents for the years 2019 and 2020. This allowed us to compare both pre-pandemic and pandemic-era visitation patterns. We examined the pandemic’s impact by integrating the difference-in-difference (DID) design with the travel cost model. Unlike previous studies, our DID design facilitated the identification of causal impacts while controlling for unobservable factors that could potentially confound the treatment effects of interest. Additionally, the travel cost model enabled us to explore the driving factors behind visitation changes and estimate the welfare changes experienced by NYC residents.

Several key findings emerged from our analysis. First, following the pandemic’s outbreak and the enforcement of New York State’s stay-at-home orders, we observed a sharp decline in visitation. This initial decline was followed by a gradual rebound in visitation that began several weeks after the initial outbreak. However, until the end of the year 2020, visitation had yet to return to pre-pandemic levels. Second, in comparison with the control year of 2019, the pandemic triggered a 94% decrease in visitation rates. The leading factors contributing to this decline include the stay-at-home order, the surge in new COVID-19 cases, and potential alterations in travel costs. Third, the pandemic induced a welfare loss exceeding $450 million in annual consumer surplus, representing a 2% loss of in the value ecosystem services provided by Central Park.

Our analysis is among the first to provide empirical evidence on how COVID-19 affected urban park recreation and residents’ welfare. By developing an innovative analytical framework that integrates mobile big data, a contemporary econometric approach, and a travel cost model along with welfare analysis, we enabled a rapid assessment of the pandemic impacts and provided a basis for valuing unpriced services provided by urban parks. Our modeling framework and analysis of the pandemic’s consequences could extend to similar events and other urban parks, which would enable policymakers and the tourism industry to make informed decisions for site management in a timely manner.

## Materials and methods

### Study site

Central Park, located in the heart of Manhattan, attracted more than 42 million visitors in 2019 and has been regarded as a model for the world’s urban parks [9]. It is the most visited urban park in the U.S. [10]. Central Park is a renowned tourist site as well as a daily activity space for residents. As in other major urban parks in metropolitan areas, a large share of the visits (70%) is made by people who live in NYC.

**Fig 1:**
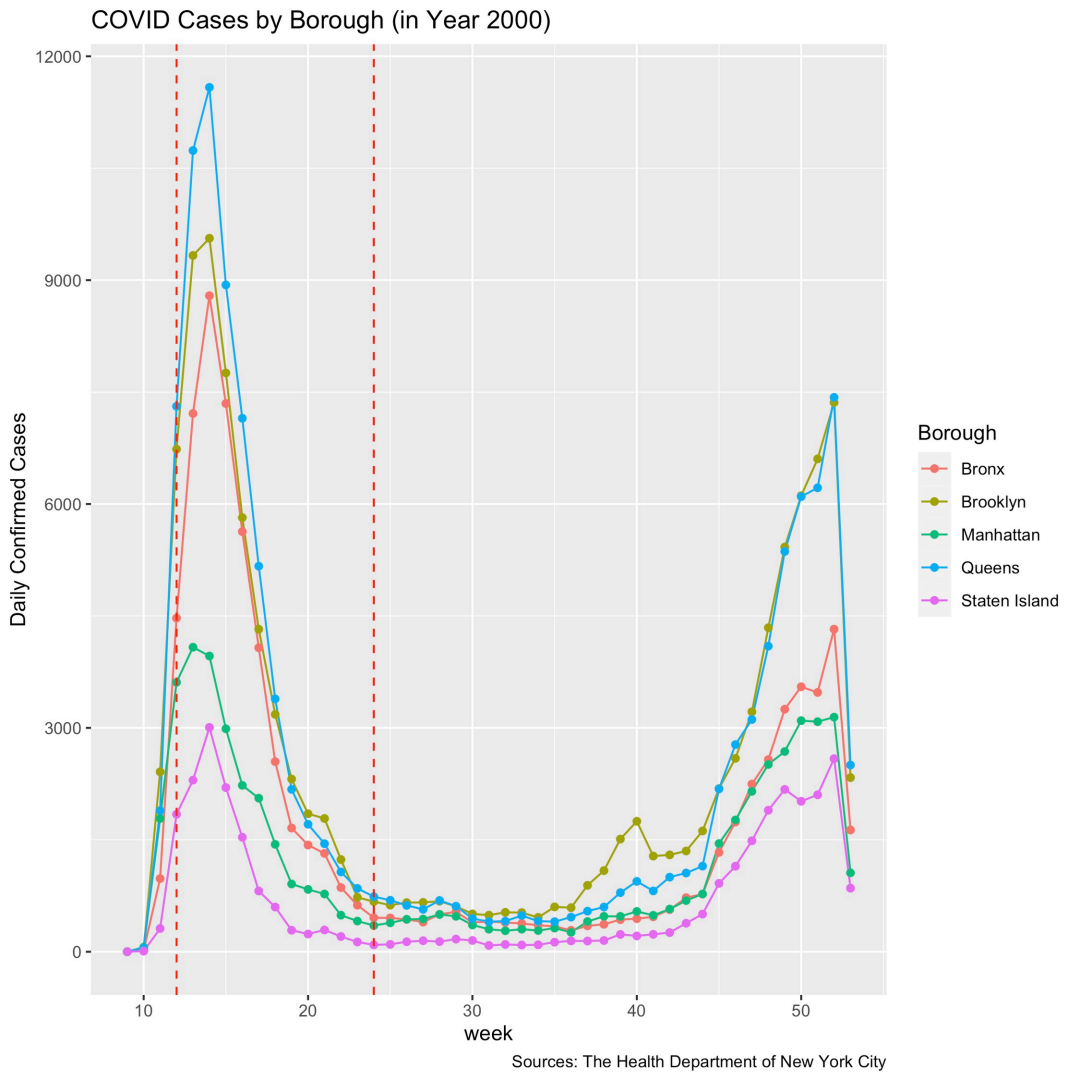
Weekly number of New Confirmed Covid Cases in New York City.

The pandemic changed park recreation in NYC dramatically. In the spring of 2020, the city suffered heavily in the early stages of the pandemic. Starting in week 11 of year 2020, the number of cases increased dramatically in the five boroughs of NYC (Fig 1). The large number of confirmed cases made NYC the world’s top COVID-19 hotspot. The park remained open after New York Governor Cuomo issued a “New York State on PAUSE” executive order (also known as “stay-at-home order”) on March 20, 2020, but access to park facilities was limited to prevent overcrowding and to promote social distancing [11].

### Cellphone data

We used cellphone data to identify weekly visits of New Yorkers to Central Park in 2019 and 2020. The data were provided by SafeGraph, a company that records the location and movement of more than 45 million mobile devices in the U.S. SafeGraph covers approximately 10% of the cellphone mobility patterns in the country. The SafeGraph data have been widely used in the literature to trace mobility patterns during COVID-19 [12–15].

In the SafeGraph dataset, a specific physical location, referred to as a Point of Interest (POI), is identified for recording foot traffic and visitor counts. Examples of POIs within Central Park include the Billy Johnson Playground, the Central Park Obelisk, the Lake at Central Park, the Ladies Pavilion, and the Thomas J. Watson Library.

SafeGraph employs a state-of-the-art visit attribution algorithm to tally the number of visitors at each POI and provides detailed information on the geometry of each POI, including location and shape. The algorithm ensures sufficient resolution to differentiate between distinct locations. To qualify as a visit to a given POI, the duration of the visit must be at least four minutes. This stipulation effectively rules out individuals who merely pass through Central Park without visiting a specific POI. There are multiple POIs within the park’s confines, representing various attractions within Central Park. We retrieved the geometry of the relevant POIs and merged it with the shapefile of Central Park to ensure that only attractions within the park are included. This process effectively excludes office buildings and other non-recreational locations adjacent to the park.

SafeGraph also provided the home block group information for each visitor, which is determined as the most common nighttime location during nighttime hours. The home location allowed us to link the visitor counts to the block group level socio-demographic information.

We adjusted the original number of visitors from the SafeGraph data, taking into account the market share of the mobile service company and the percentage of U.S. citizens owning smartphones, to reflect the total number and trends of the visitors. Specifically, the number of visitors is calculated using the following equation:

$$V_{t}=N_{t}/M$$ (1)

Where *V*_*t*_ is the adjusted number of visitors in each week *t*, *N*_*t*_ is the original number of visitors from SafeGraph data, *M* is the adjustment indictor, which is a multiplier of market share of mobile service company (10%) and the percentage of U.S. citizens with smart phones (81%). Compared with the total number of visitors in 2019, our dataset covers approximate 11% of the NYC visitors to Central Park [9].

### Econometric model

We used a quasi-experimental design to identify visitation impacts. We incorporated a difference-in-differences (DID) design in a recreation-demand model to control for unobservable factors that might confound the treatment effects of interest.

DID is typically used to estimate the effect of a specific treatment by comparing the changes in outcomes between control and treatment groups. In our case, pandemic is the treatment; year 2019 is the control group, and year 2020 is the treatment group; weeks 1–11 are the pre-treatment periods, and weeks 12–52 are the post-treatment periods. We included a rich set of fixed effects and control variables to consider other driving factors of visitation changes.

The key identification assumptions in the DID design are parallel trends and no anticipation [16]. The parallel trends assumption implies that, in the absence of the pandemic, the visitation patterns of the control group (year 2019) and the treatment group (year 2020) would have been parallel. The no anticipation assumption necessitates that visitation patterns in pre-treatment periods do not affect the treatment status in the post-treatment periods. We used the Wald test to examine the parallel trend assumption [17]. The testing results yielded a p-value of 0.44, indicating a failure to reject the null hypothesis that linear trends are parallel. The unforeseen nature of the pandemic also supports the fulfillment of the no anticipation assumption.

We used a classical zonal travel cost demand model (ZTCM) to assess the number of visitors originating from various zones, and to study the changes in welfare due to COVID-19 [18–20]. We define neighborhood tabulation areas (NTAs) as our zones. NTAs are designations used by the New York Department of City Planning for small area population projections. Within New York City, there are 195 NTAs with a minimum population of 15,000 in each area. The existence of a substantial number of zero values made it hard to estimate the zonal travel cost model at the block group level. Thus, we chose to estimate the model at an aggregate level.

The ZTCM establishes a relationship between visitation rate and a set of explanatory variables believed to influence visitation:

$${VR}_{ist}=f({TC}_{ist},S_{ist})$$ (2)

where *VR*_*ist*_ is the visitation rate of zone *i* in year *s* of week *t*, which reflects the number of visitors from zone *i* per 1000 population; *TC*_*ist*_ is the travel cost from zone *i* to Central Park, and *S*_*ist*_ represents policy and socio-economic variables.

The travel cost of a visit is the round-trip travel costs to Central Park from visitors’ NTAs. To calculate travel costs, it is essential to assume a specific transportation mode. Considering the unique characteristics of NYC, we have selected walking and taxi rides as the modes of transportation for calculating travel costs. If walking is the chosen mode of transportation, travel costs will primarily consist of the monetary value of time spent via walking. Conversely, if traveling by taxi, the travel costs will be a combination of the taxi fare and the monetary value of the time spent during the ride. We used the walking mode as an approximation to calculate travel costs related to both walking and biking, and the taxi mode as an approximation for calculating travel costs associated with for-hire vehicles as well as driving one’s own car.

In our calculations, we used an 800-meter distance (approximately 0.5 miles) as the threshold for determining the mode of transportation. If the distance to be covered is less than 800 meters, individuals are assumed to choose walking; otherwise, we assume they are likely to opt for a taxi. The rationale behind selecting 800 meters as the threshold is rooted in a combination of findings from existing literature and the particular context of NYC. Previous studies have suggested that 400 meters (equivalent to 0.25 miles or a 5-minute walk) is the distance that the average American is willing to walk before deciding to drive [21]. However, another study also indicates that it is not uncommon for individuals to walk distances greater than 0.25 miles [22]. Given that New Yorkers frequently walk as a means to reach specific destinations, an 800-meter (0.5 miles) threshold was deemed appropriate for our analysis.

We took into account the monetary value of time to reflect the opportunity costs, i.e., people would weigh the trade-offs between leisure and work in their recreational decisions [7]. We calculated the monetary value of time spent on travel based on per-minute per capita income data from the census survey and calculated the taxi fare using NYC’s taxi rates [23,24]. Travel distance and time were calculated using OpenStreetMap, an open-source tool that offers functionalities similar to Google Maps.”

We chose the log-log form as the model specification, since the AIC and BIC favored the nonlinear form, and the coefficient of interests were robust to model specifications.

#### Overall impacts of the pandemic

To explore the overall impacts of the pandemic on visitation patterns, we specify the following econometric model:

$$ln({VR}_{ist})=\alpha +\beta {ln(TC}_{ist})+\sigma X_{ist}+\gamma {Year20}_{s}+\theta {TreatWeek}_{t}+\delta ({Year20}_{s}{*TreatWeek}_{t})+{\eta X}_{ist}+\varphi T_{t}+\varepsilon_{ist}$$ (3)

where *ln*(*VR*_*ist*_) is the log form of visitation rate, ln (*TC*_*ist*_) is the log form of the average travel cost to visit Central Park; ***X***_***ist***_ is a vector of socio-demographic characteristics, weather, and holiday variables including NTA-specific median household income, median age, and percentage of people with high school diplomas; borough-specific average precipitation and average daily maximum temperature; and weeks with national holidays. We obtained year-variant socio-demographic data from the American Community Survey 5-year estimate on the Census Block Group level, and then aggregate block group level data to NTAs [23]. Covid and weather-related data are constructed at borough level [25]. We obtained daily weather estimates from GRIDMET and then aggregate daily level weather data by week. *Year*20_*s*_ is our treatment group. It takes a value of one if the visitation occurs in 2020 and zero otherwise. *Treatweek*_*t*_ is our treatment period; it takes a value of one if visits occured after week 12 and zero otherwise; **T** represents monthly fixed effects; and *ε*_*ist*_ an error term.

Our coefficient of interest is *δ* of term (*Year*20_s_*TreatWeek_*t*_), which measures the overall impact of the pandemic on park visitation with the control of counterfactuals. Specifically, *δ* represents the average effects of the “after week 12 period in 2020” relative to the “after week 12 period in 2019” with the control of any background differences in visitation between 2019 and 2020. By background differences, we mean any effects shared by *all weeks* in 2020 versus 2019 in the absence of the pandemic, which is captured by *γ*. An example of this might be higher visitation realized in 2020 due to major park improvements. *θ* represents the average differences between the post-treatment periods (week 1–11) and the pre-treatment periods (week 12–52) in the absence of the pandemic. It is important to keep in mind that our term (*Year*20_s_*TreatWeek_*t*_) picks up all effects due to the pandemic–individual choice, regulations, public health information, etc.

#### Mechanisms of visitation changes

We adapted the model specifications based on Eq (3) to investigate the factors driving changes in visitation. These factors include lockdown policy, public health information, and potential income fluctuations.

To isolate the effects of policy restrictions and public health information, we included a policy variable (*P*_*st*_) and number of new cases (*NewCase*_*ist*_) into the regression. The policy variable *P*_*st*_ is defined based on the duration of stay-at-home order: it has a value of 1 during stay-at-home periods (weeks 12–24 in year 2020) and have a value of 0 at all other times. We rewrote Eq (3) as follows:

$$ln({VR}_{ist})=\alpha +\beta {ln(TC}_{ist})+\sigma X_{ist}+\gamma {Year20}_{s}+\theta {TreatWeek}_{t}+\delta ({Year20}_{s}{*TreatWeek}_{t})+\rho P_{st}+\mu {NewCase}_{ist}+{\eta X}_{ist}+\varphi T_{t}+\varepsilon_{ist}$$ (4)

We are still interested in the treatment effect *δ*, albeit the underlying interpretations differ across models. In this case, *ρ* represents the impact of the stay-at-home order on park visitation, reflecting people’s compliance with restrictions, and their initial responsiveness to COVID-19 risk. The sign and significance of *μ* indicate the impact of public health information on recreational behavior, shedding light on the visitors’ responsiveness to COVID-19 risks. Finally, *δ* would capture the remaining impacts on visitation patterns, which might relate to behavioral changes and other factors.

It is possible that stay-at-home order and the pandemic would bring significant changes to the impact of travel costs by changing residents’ income levels, transportation modes, and behaviors. Thus, we further tested the heterogeneity of the travel costs impacts, in which:

$$ln({VR}_{ist})=\alpha +\beta {ln(TC}_{ist})+\sigma X_{ist}+\gamma {Year20}_{s}+\theta {TreatWeek}_{t}+\delta ({Year20}_{s}{*TreatWeek}_{t})+\rho P_{st}+\mu {NewCase}_{ist}+\pi P_{st}*{ln(TC}_{ist})+\tau {NP}_{st}*{ln(TC}_{ist})+{\eta X}_{ist}+\varphi T_{t}+\varepsilon_{ist}$$ (5)

where *NP*_*st*_ is a dummy variable which indicates the post-pandemic periods without stay-at-home orders. A significant *π* indicates that the effect of travel costs is different during stay-at-home periods and the non-stay-at-home periods, and a significant *τ* indicates the heterogeneity impacts of travel costs would last longer than the stay-at-home periods.

### Valuation of pandemic impacts

In this paper, we estimated economic value changes based on visitors’ welfare changes.

According to economic theory, for park visitors, if expected benefits are greater than the travel costs, visitors would take the trip; otherwise, visitors would not take the trip. When a trip is taken, consumer surplus (CS) would arise through the differences of expected benefits and travel costs. Changes in visitation patterns will result in changes in consumer surpluses, which would reflect the shifts in societal welfare. Quantifying the pandemic-induced welfare changes can provide valuable insights into the magnitude of the impacts on social well-being.

We calculated consumer surplus of observed visitors for three separate periods in both year 2019 and 2020—pre-pandemic weeks (weeks 1–11), post-pandemic weeks with stay-at-home order (weeks 12–24), and post-pandemic weeks without stay-at-home order (weeks 25–52) [26]. Our calculations utilize the estimation results of Eq (5) and are based on the following formula:

$$CS=-\frac{e^{\hat{\beta_{0}}}}{\hat{\beta_{1}}+1}\sum_{i}(\frac{{pop}_{i}}{1000})({{TC}_{i})}^{\hat{\beta_{1}}+1}$$ (6)

Where

$$\left\{ \begin{array}{ll} {\hat{\beta }}_{0}=\alpha +\sigma X_{ist}+\gamma {Year20}_{s}+\theta {TreatWeek}_{t}+\delta ({Year20}_{s}{*TreatWeek}_{t})+{\eta X}_{ist}+\varphi T_{t} & if\;t\epsilon [0,1]\\ {\hat{\beta }}_{0}=\alpha^{\prime }+\sigma X_{ist}+\gamma {Year20}_{s}+\theta {TreatWeek}_{t}+\delta ({Year20}_{s}{*TreatWeek}_{t})+\rho P_{st}+\mu {NewCase}_{ist}+{\eta X}_{ist}+\varphi T_{t} & if\;t\epsilon [12,24]\\ {\hat{\beta }}_{0}=\alpha^{\prime \prime }+\sigma X_{ist}+\gamma {Year20}_{s}+\theta {TreatWeek}_{t}+\delta ({Year20}_{s}{*TreatWeek}_{t})++\mu {NewCase}_{ist}+{{\eta X}_{ist}+\varphi }^{\prime \prime }T_{t} & if\;t\epsilon [25,52]\\ {\hat{\beta }}_{1}=\beta & if\;t\epsilon [0,11]\\ {\hat{\beta }}_{1}=\beta +\pi P_{st} & if\;t\epsilon [12,24]\\ {\hat{\beta }}_{1}=\beta +\tau {NP}_{st} & if\;t\epsilon [25,52] \end{array}\right.$$

For each period, we take the average values of *Year*, *TreatWeek*, ***X*, *T*, *P*, *NewCase*** in each zone in the calculation. It is also worth noting that CS in Eq (6) reflect aggregated consumer surpluses in each period. To compare across periods, we also calculated per-week consumer surpluses of all NYC visitors in each period, then aggregated annual consumer surpluses are calculated for year 2019 and year 2020 to reflect annual economic impacts.

## Results

### Descriptive analysis of COVID-19’s impact on visitation

**Fig 2:**
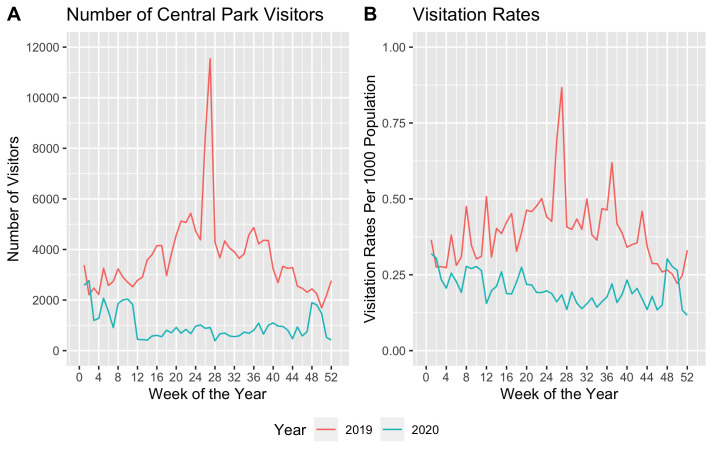
Weekly patterns of number of visitors and visitation rates of central park. Note: Panel A presents trends of number of visitors from SafeGraph data (*N*_*t*_), and panel B presents trends of visitation rates per 1000 population (*VR*_*ist*_).

Fig 2 illustrates patterns of visitors and visitation rates. We observed a similar number of visitors in weeks 1–11 in 2019 and 2020, which suggests parallel visitation patterns between the two years in the absence of the pandemic (Fig 2, Panel A). In 2019, the number of visitors increased thereafter, peaking in weeks 26–30, which exhibits the same pattern as the Central Park Conservancy’s data [27]. Conversely, in 2020, we observed a sharp decrease of visitors in week 12. The decrease is no doubt due to the fast-growing number of COVID-19 cases and “stay-at-home” order issued on March 20. NYC was logging 2,000 cases per day during week 12, and the number grew thereafter. The stay-at-home order banned all non-essential gatherings. As shown, after the initial decline, there is a gradual rebound. By week 48, visitation in 2020 had returned to its 2019 level, but then again began to decline in the final weeks of the year. These data, based on visual inspection, suggest a large COVID impact on Central Park visitation.

In contrast to the sharp decline observed in panel (a), the variations in panel (b) have been notably more gradual since the brunt of the pandemic. The changes in the visitation rate could be influenced by both the fluctuations in the number of visitors and the total population within each zone. The pandemic led to a net outmigration among New Yorkers, which reduced the population base and accounts for the gentler changes in panel (b) [28]. Moreover, the differences between these two panels further reinforce the importance of Central Park: individuals who are regular visitors to Central Park demonstrate a lower likelihood of relocating, emphasizing the park’s integral role in helping the community manage the pressures of the pandemic.

Additionally, we provide summary statistics for visitation rates, travel costs, sociodemographic data, weather conditions, and other control variables in Table 1. These results are presented separately for each of six periods, aligned with the distinct phases of the pandemic and corresponding policy responses.

**Table 1:** Descriptive statistics of key variables.

Variable	Year 2019			Year 2020		
	*Week 1–11*	*Week 12–24*	*Week 25–52*	*Week 1–11*	*Week 12–24*	*Week 25–52*
*Visitation Rates* *per 1000 population*	0.23 (0.70)	0.34 (0.91)	0.33 (0.85)	0.18 (0.40)	0.05 (0.21)	0.10 (0.25)
*Travel cost ($)*	70.69 (34.15)	71.48 (34.29)	71.64 (34.77)	71.53 (34.88)	70.18 (34.03)	71.03 (34.06)
*Daily Confirmed New Cases*	0 (0)	0 (0)	0 (0)	12 (41)	3,836 (3,459)	1,708 (2,004)
*Median household income ($)*	75,665 (32,145)	75,665 (32,145)	75,665 (32,145)	75,997 (31,606)	75,997 (31,606)	75,997 (31,606)
*Median age*	38.16 (4.53)	38.16 (4.53)	38.16 (4.53)	38.24 (4.59)	38.24 (4.59)	38.24 (4.59)
*Percent of high school degrees (%)*	81.8% (9.5%)	81.8% (9.5%)	81.8% (9.5%)	81.9% (9.3%)	81.9% (9.3%)	81.9% (9.3%)
*Precipitation (mm)*	3.07 (1.66)	4.39 (2.39)	3.78 (3.55)	1.67 (1.43)	3.07 (2.40)	3.74 (3.04)
*Maximum daily temperature (degrees F)*	41.73 (4.68)	65.59 (8.17)	69.03 (16.37)	47.68 (5.75)	64.32 (9.64)	70.34 (15.37)
*Week with national holidays*	0.27 (0.45)	0.07 (0.27)	0.21 (0.41)	0.27 (0.45)	0.07 (0.27)	0.18 (0.38)

Note: This table presents the mean and standard deviation (in parentheses) of the variables of interest for each period. Weeks 1–11 represent the pre-treatment weeks, while weeks 12–52 represent the post-treatment weeks. In 2020, a stay-at-home order was in effect from weeks 12 to 24. Visitation Rates per 1000 population is the dependent variable in our econometric model.

Overall, the summary statistics underscore substantial changes in visitation rates between the control year (year 2019) and the treatment year (year 2020). Compared to the first 11 weeks, in a year like 2019, visitation rates would increase in weeks 12–24 and in weeks 25–52 due to seasonal effects and better weather conditions. However, we observe a large decrease in average visitation rates for year 2020. When compared with the first 11 weeks in 2020, the average visitation rates decreased 83% for the post-pandemic period with stay-at-home policy (weeks 12–24) and decreased 70% for the post-pandemic period without stay-at-home policy (weeks 27–52).

Turning to explanatory variables in the regressions, the summary statistics suggest a large variation of travel costs, household income, age, and education across study zones; but the differences across study periods are minimal. Given the spread of the pandemic, it was not surprising to see the dramatic increase of confirmed new cases in weeks 12–52 of year 2020. We also observed spatial and temporal change in weather conditions. Collectively, the descriptive evidence suggests that controlling for a number of observed and unobserved time-varying changes is likely needed to identify the impact of the pandemic on recreational demand.

#### Econometric analysis of overall impacts and associated mechanisms

Based on the econometric models, we present the estimated coefficients for three separate models in Table 2. The coefficients in bold are our main focus. Across all three models, we observed significant and negative coefficients, indicating that the pandemic has markedly decreased visitation rates. Moreover, t-tests revealed substantial differences between the bolded coefficients across the models.

**Table 2:** Estimation results of DID design.

Variable	Model 1	Model 2	Model 3
*ln(Travel cost)*	-1.930*** (0.030)	-1.937*** (0.030)	-1.766*** (0.034)
*Treatweek (= 1)*	-0.611*** (0.139)	0.171 (0.143)	0.167 (0.143)
*Year20 (= 1)*	-0.387*** (0.089)	-0.309*** (0.089)	-0.307*** (0.089)
***Treatweek*** * ***Year20 (= 1)***	**-2.796** *** **(0.099)**	**-2.305** *** **(0.105)**	**-1.069** *** **(0.287)**
*Stay-at-home Order (= 1)*	-	-1.964*** (0.097)	-0.447 (0.487)
*Daily Confirmed New Cases*	-	0.00002 (0.00001)	0.00003** (0.00001)
*Stay-at-home Order (= 1)* * *ln(Travel cost)*	-	-	-0.680*** (0.107)
*Pandemic non-stay-at-home Order (= 1)* * *ln(Travel cost)*	-	-	-0.304*** (0.068)
*Median household income*	0.0001*** (1.05 ·10^−6^)	0.0001*** (1.03·10^−6^)	0.00001*** (1.02·10^−6^)
*Median age*	0.031*** (0.005)	0.031*** (0.005)	0.031*** (0.005)
*Percent of high school degree*	0.313 (0.402)	0.332 (0.395)	0.313 (0.395)
*Precipitation*	-0.004 (0.007)	-0.024*** (0.007)	-0.024*** (0.007)
*Maximum daily temperature*	0.056*** (0.004)	0.038*** (0.005)	0.038*** (0.005)
*Week with national holidays (= 1)*	0.230*** (0.057)	0.318*** (0.057)	0.318*** (0.057)
*Constant*	-0.416 (0.344)	0.034 (0.117)	-0.368 (0.348)
*Monthly Fixed Effect*	Yes	Yes	Yes
*Observations*	21,944	21,944	21,944

Note: This table presents estimated results of DID design following Eqs (3), (4), and (5), respectively for models (1), (2), and (3). Robust standard errors are reported in parentheses.

*** p<0.01

** p<0.05

* p<0.1.

The bolded coefficient in the first column (Model 1, based on Eq 3) encapsulates the aggregate effects of the pandemic. This includes the impacts of the stay-at-home order, public health information, voluntary avoidance behavior, changes to recreation sites, among others. In the second column (Model 2, based on Eq 4), we isolated the impact of the stay-at-home order and public health information. Thus, the bolded coefficient in this column reflects the changes instigated by other aspects of the pandemic. In the third column (Model 3, based on Eq 5), we incorporated the interaction of the pandemic and travel costs into the regression. Therefore, the bolded coefficient in this column signifies the residual effects linked to the pandemic.”

We calculated the changes in visitation rate based on the estimated coefficients, where the percentage change is given by (1−*e*^*δ*^)*100%. Overall, the pandemic reduced the visitation rate by 94% (model 1). With the consideration of stay-home-order and potential changes in travel costs, the pandemic still brought a 66% impacts to the visitation rates (model 3).

The coefficients for travel costs in all three models are negative and significant, suggesting that an increase in travel costs results in a decrease in recreational demand. A comparison utilizing t-tests reveals no significant differences between Model 1 and Model 2. However, there are notable differences between Model 1 and Model 3, as well as between Model 2 and Model 3. These differences are likely due to the inclusion of additional interaction terms in Model 3.

Based on Model 3, overall, a 1% increase in travel costs corresponds to a 1.77% decrease in visitation rates. During the weeks when the stay-at-home order was in effect, a 1% increase in travel costs led to a more substantial decline in visitation rates of 2.45%. This larger change could be attributed to the suspension of public transportation and implementation of restrictive policies. Notably, the pandemic continued to significantly impact visitation rates even in the absence of a stay-at-home order. During pandemic weeks without such an order, a 1% increase in travel costs caused a 2.07% drop in visitation rates. These residual impacts may be linked to shifts in work modes, such as ongoing remote work, and alterations in time costs.

There are other factors that can affect recreational demand. Based on model 3, we find a significant and positive relationship between the number of new COVID cases per week and visitation rates, which sheds lights on the positive impacts of urban parks, i.e., outdoor recreation to urban parks could serve as an important and relatively safe escape for people to cope with the stresses of the pandemic. Additionally, we found that positive and significant coefficients for median household income and median age, suggesting that zones with higher income levels and an older population, were associated with a higher demand to visit Central Park. Weather also played a key role—we found that higher temperatures increased visitation rates, while precipitation decreased them. Furthermore, we found that weeks with holidays generated more visits, which highlights the trade-offs between leisure and work.

### Economic and welfare impacts

Using coefficients from the econometric analysis, we calculated per week consumer surplus for weeks in three stages: pre-pandemic weeks (weeks 1–11), post-pandemic weeks with stay-at-home policy (weeks 12–24), and post-pandemic weeks without stay-at-home policy (weeks 25–52). We then aggregated weekly consumer surplus to annual levels for year 2019 and year 2020.

The consumer surplus estimates are presented in Table 3. During the pre-pandemic weeks, control year and treatment year demonstrate similar welfare impacts per week. On average, in the first 11 weeks of the year, consumer surplus from visitation to Central Park is around $5 million per week. In year 2019, consumer surplus increases after week 12 and reaches to more than $11 million per week. The increase would relate to better weather conditions and multiple national holidays.

**Table 3:** Estimation of Consumer Surplus (CS).

	Year 2019	Year 2020
*Per-week CS (Weeks 1–11)*	$4.9 million [$2.2 million, $7.5 million]	$4.5 million [$1.9 million, $7.1 million]
*Per-week CS (Weeks 12–26)*	$11 million [$3.2 million, $19 million]	$0.2 million [$0.1 million, $0.4 million]
*Per-week CS (Weeks 27–52)*	$12 million [$3.8 million, $20 million]	$0.8 million [$0.4 million, $1.5 million]
Annual CS	$532 million [$173 million, $892 million]	$74 million [$23 million, $125 million]

Note: Numbers in brackets are 95% confidence intervals.

But the pandemic changed everything. Comparing the post-pandemic periods with the scenario with no-pandemic, our result indicates over $11 million decrease of consumer surplus per week. Overall, when comparing the treatment year with the control year, our results indicated more than $450 million decrease in total welfare. The value of urban ecosystem services provided by Central Park is is estimated more than $23,800 million per year [29]. Thus, COVID-19 caused 2% losses of values in year 2020.

## Discussion and conclusion

Understanding and assessing the impacts of the pandemic on outdoor recreation can provide valuable insights for the management of recreation areas [30]. Using the iconic Central Park as a study site, our analysis benefits from a large cellphone mobility dataset that enables us to explore how the COVID-19 pandemic changed New Yorkers’ engagement with Central Park and impacted the economic value they derived from those interactions. Our results highlight the enormous effect the COVID-19 pandemic have had on the recreation activities of local residents, with a 94% visitation decrease and a reduction of over $450 million in annual consumer surplus. While some rebound effects were observed after a few months, the loss remained significant.

As one of the pioneering empirical studies assessing the economic impact of COVID-19 on urban parks, our findings shine a light on the substantial, yet often overlooked, welfare loss triggered by the pandemic. The decline in visitation and consumer surplus not only indicates an economic loss, but also hints at a larger issue–the loss of exercise and relaxation opportunities that could have mitigated the stress induced by the pandemic. Although we may not be on the brink of another pandemic, the likelihood of facing similar crises in the future is present. Such events highlight the significance of public open spaces for exercise and relaxation away from home. In summary, our study offers valuable insights for urban park management and suggests considerations for potential responses to sudden, large-scale disruptions akin to the pandemic.

We perceive the integration of mobile big data with the conventional travel cost method as a promising opportunity in urban park management, particularly for crisis impact estimation. The framework offers an applicable and cost-effective way of monitoring the recreational value of outdoor sites over an extended period and across large spatial coverage. Being the largest city and one of the greenest cities in the U.S., New York City is actively working on enhance park facilities and amenities. A cost-effective way to monitor the recreational values on a regular basis would be helpful in analyzing travel behavior changes and provide scientific references for future park investments [31,32].

It is worth highlighting limitations of our study. First, the estimation of travel costs could be further refined. Our current data constraints limit our ability to completely encompass the shifts in travel behaviors and costs that occurred during the pandemic. Second, due to Central Park’s considerable size and the scattered distribution of POIs, generating an accurate estimation of park usage is a substantial challenge. The potential inaccuracy of these estimations may introduce biases into the calculation of consumer surplus. Third, our analysis is solely based on local residents. Consequently, our estimation of the welfare loss likely represents a lower-bound figure. Lastly, the impact of pandemic-induced migration on recreation demand remains an open question. Future research should delve into the spatially varying impacts and related welfare changes to provide a more comprehensive understanding.
